# Induction of cell wall phenolic monomers as part of direct defense response in maize to pink stem borer (*Sesamia inferens* Walker) and non-insect interactions

**DOI:** 10.1038/s41598-021-93727-2

**Published:** 2021-07-20

**Authors:** P. Lakshmi Soujanya, J. C. Sekhar, C. V. Ratnavathi, Chikkappa G. Karjagi, E. Shobha, S. B. Suby, K. R. Yathish, N. Sunil, Sujay Rakshit

**Affiliations:** 1 Winter Nursery Centre, ICAR-Indian Institute of Maize Research, Rajendranagar, Hyderabad, 500030 India; 2 ICAR-Indian Institute of Millets Research, Rajendranagar, Hyderabad, 500030 India; 3 Unit Office, ICAR-Indian Institute of Maize Research, Delhi Unit, Pusa Campus, New Delhi, 110 012 India; 4 ICAR-Indian Institute of Maize Research, PAU Campus, Ludhiana, Punjab 141004 India

**Keywords:** Entomology, Herbivory

## Abstract

Pink stem borer (PSB) causes considerable yield losses to maize. Plant–insect interactions have significant implications for sustainable pest management. The present study demonstrated that PSB feeding, mechanical wounding, a combination of mechanical wounding and PSB regurgitation and exogenous application of methyl jasmonate have induced phenolic compound mediated defense responses both at short term (within 2 days of treatment) and long term (in 15 days of treatment) in leaf and stalk tissues of maize. The quantification of two major defense related phenolic compounds namely *p*-Coumaric acid (*p*-CA) and ferulic acid (FA) was carried out through ultra-fast liquid chromatography (UFLC) at 2 and 15 days after imposing the above treatments. The *p*-CA content induced in leaf tissues of maize genotypes were intrinsically higher when challenged by PSB attack at V3 and V6 stages in short- and long-term responses. Higher *p*-CA content was observed in stalk tissues upon wounding and regurgitation in short- and long-term responses at V3 and V6 stages. Significant accumulation of FA content was also observed in leaf tissues in response to PSB feeding at V3 stage in long-term response while at V6 stage it was observed both in short- and long-term responses. In stalk tissues, methyl jasmonate induced higher FA content in short-term response at V3 stage. However, at V6 stage PSB feeding induced FA accumulation in the short-term while, wounding and regurgitation treatment-induced defense responses in the long-term. In general, the resistant (DMRE 63, CM 500) and moderately resistant genotypes (WNZ ExoticPool) accumulated significantly higher contents of *p*-CA and FA content than susceptible ones (CM 202, BML 6) in most of the cases. The study indicates that phenolic mediated defense responses in maize are induced by PSB attack followed by wounding and regurgitation compared to the other induced treatments. Furthermore, the study confirmed that induced defense responses vary with plant genotype, stage of crop growth, plant tissue and short and long-term responses. The results of the study suggested that the Phenolic acids i.e.* p*-CA and FA may contribute to maize resistance mechanisms in the maize-PSB interaction system.

## Introduction

The pink stem borer (PSB), *Sesamia inferens* Walker is one of the major insect pests in the winter maize which results 25.7–78.9 percent yield losses^1^.The larvae feed inside the leaf sheath in groups and subsequently bore into the central shoot resulting in the formation of dead hearts. The larvae form circular-shaped tunnels inside the stem and exit holes on the stem surface. PSB can be managed by spraying chemical insecticides but indiscriminate use of chemicals raises concerns over residues, insecticide resistance and environmental pollution. Understanding the mechanisms of Host Plant Resistance (HPR) can provide an opportunity to manage PSB in the long-run. HPR is an economically viable, environmentally friendly, and sustainable strategy and is one of the most effective components of integrated pest management (IPM) modules.

Different kinds of stimuli like mechanical wounding, herbivore attack, application of insect regurgitation, phytohormones etc. results in a change in the biochemical constituents of plants^2,3^. Plants recognize such external stimuli as herbivore attack and induces defense responses against herbivores^4^. HPR is the result of such plant defenses (direct or indirect) and it continually evolves in response to insect attack. The alteration in the biochemical composition of plants especially related to defensive responses affects the fitness and behavior of insects^5^. The direct defense responses include the change in morphological traits, production of secondary metabolites that either kills or slows the development of herbivores^6,7^, while indirect defenses include the release of volatile organic compounds that attract natural enemies of insect-pest(s). Understanding the plant–insect interactions is of utmost importance for developing effective pest management approaches. Direct damage to the plant or exposure to volatiles results in induced resistance^8^. Induced defense responses compromise less plant fitness and are more durable as compared to constitutive defense mechanisms^9^. PSB with limited host-range cannot adapt easily and quickly to the variety of plant toxic compounds. Therefore, it is hypothesized that the induced resistance could be more successful for PSB management under field conditions. While feeding on a plant, insects often regurgitate which contains different elicitors and the role of different elicitors in plant defense-related responses have been demonstrated. Besides, Jasmonic acid an important phytohormone derived from linolenic acid through the octadecanoid pathway is also known to activate the expression of both direct and indirect defense responses upon insect attack^10^. Through quantification of secondary metabolites, it is possible to measure induced direct defense response by the host-plant. The previous study has demonstrated that cell wall-bound phenolic acids, i.e.* p*-coumaric acid (*p*-CA) and ferulic acid (FA) may impart resistance to PSB in maize^11^. The present study was undertaken to study whether the resistance was inducted in response to plant–insect and/or non-insect interactions. Understanding the nature of plant defensive traits will help in better protection from insect herbivores and also for optimizing sustainable maize production. Therefore, the present study tested the following hypotheses (i) PSB feeding, mechanical wounding, a combination of mechanical wounding and PSB regurgitation, and exogenous application of methyl jasmonate (MeJA) would induce plants for defense responses; (ii) induced defense responses vary with stages of crop growth, plant tissue, and time (short and long-term); (iii) the maize genotypes respond differently to the induced treatments based on the degree of resistance to PSB.

## Results and discussion

### The response of lines to PSB infestation under field conditions

The data on the Leaf Injury Rating (LIR) score of five genotypes screened against PSB under artificial infestation in field conditions was presented in Table 1. The field screening confirmed the earlier report that DMRE 63 (2.9 ± 0.18) and CM 500 (3.0 ± 0.20) were resistant with minimum LIR. WNZ Exotic Pool (4.3 ± 0.18) was moderately resistant with medium LIR and CM 202 (7.7 ± 0.26) and BML 6 (7.3 ± 0.22) were susceptible with LIR score of > 7 against PSB infestation. Based on the categorization of resistance/susceptibility, the genotypes were selected for the quantification of cell-wall bound phenolics in leaf and stalk tissues after exposure to different induced treatments.

**Table 1:** Leaf Injury Rating of maize genotypes based on 1–9 scale by pink stem borer.

Inbred line	Pedigree	Pest reaction	LIR (1–9 Scale)
DMRE 63	CM 500 SEL	Resistant	2.9 ± 0.18^d^
CM 500	Antigua group I	Resistant	3.0 ± 0.20^d^
WNZ Exotic Pool	WNZPBTL1/2/3/4/5/6/7/8/9####	Moderately resistant	4.3 ± 0.18^c^
CM 202	C121E	Susceptible	7.7 ± 0.26^a^
BML 6	SRRL 65-B96-1-1-2-#-2-2-1-** ⊗ **-1-1-** ⊗ **b-** ⊗ **b	Susceptible	7.3 ± 0.22^b^
LSD (p = 0.05)			0.31

Each value represents the mean ± SEm of four replications. Means within a column followed by different letters are significantly different (LSDTest p = 0.05).

### Induced defense response

The present study hypothesized that feeding by PSB larva, mechanical wounding, a combination of wounding and regurgitation, and exogenous application of methyl jasmonate can induce the accumulation of cell wall-bound phenolics in leaf and stalk tissues of maize, thereby imparting resistance. Therefore, the changes in cell wall-bound phenolics i.e. *p*-CA, and FA content was quantified at two stages namely V3 and V6 in leaf and stalk tissues of treated and untreated plants to elucidate the short- (2 DAT) and long-term responses (15 DAT).

#### Changes in p-CA content after imposition of different treatments in leaf tissues

**Figure 1:**
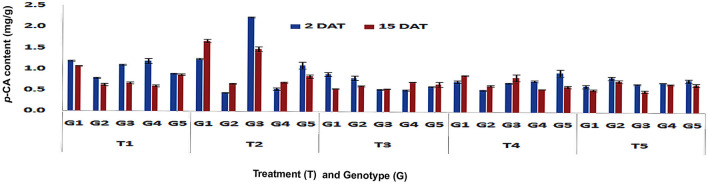
Changes in *p*-CA content in leaf tissues of maize genotypes to induced treatments in short (2 DAT) and long (15 DAT) term responses on the imposition of treatments at V3 stage (G1: DMRE 63; G2: CM 500; G3: WNZ Exoticpool; G4: CM 202; G5: BML 6; T_1_: Control; T_2_: PSB feeding; T_3_: Wounding; T_4_: Combination of wounding plus regurgitation; T_5_: Exposure to methyl jasmonate).

**Figure 2:**
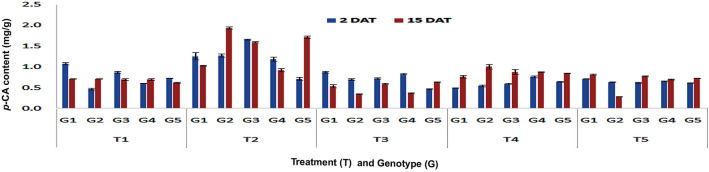
Changes in *p*-CA content in leaf tissues of maize genotypes to induced treatments in short (2 DAT) and long (15 DAT) term responses on the imposition of treatments at V6 stage (G1: DMRE 63; G2: CM 500; G3: WNZ Exoticpool; G4: CM 202; G5: BML 6; T_1_: Control; T_2_: PSB feeding; T_3_: Wounding; T_4_: Combination of wounding plus regurgitation; T_5_: Exposure to methyl jasmonate).

The feeding by PSB larvae on V3 stage plants significantly increased the accumulation of *p*-CA content in the leaves of moderately resistant genotype WNZ ExoticPool in both short-term (2.23 mg/g; Fp < 0.0001, LSD = 0.06) and long-term (1.48 mg/g; Fp < 0.0001, LSD = 0.16), whereas in the resistant genotype DMRE 63 only long-term response was significant (1.67 mg/g; Fp < 0.0001, LSD = 0.16) (Fig. 1). Whereas the PSB feeding onV6 stage plants, significantly increased the *p*-CA content in leaves of resistant genotypes namely DMRE 63 (1.26 and 1.03 mg/g) and CM 500 (1.28 and 1.94 mg/g) and moderately resistant genotype WNZ ExoticPool (1.66 and 1.60 mg/g) in short-(Fp < 0.0001, LSD = 0.06) and long-term responses (Fp < 0.0001, LSD = 0.29), respectively (Fig. 2). Similarly in the susceptible genotypes namely CM 202 (1.19 mg/g) and BML6 (1.72 mg/g) also showed induced defense responses with increased *p*-CA content in leaves upon PSB feeding in the short-term and long-term, respectively. This is in contrary to expectations, however, the *p*-CA content in resistant genotypes was 1.14 fold higher as compared to susceptible ones. This suggests that feeding by PSB strongly induced defense responses in leaves of resistant and moderately resistant genotypes both in short- (2 DAT) and long-term (15 DAT). Even though the increased *p*-CA content was less in susceptible genotypes as compared to resistant genotypes, it has been speculated why increased defense responses were also observed in susceptible genotypes. One explanation of higher defense responses when exposed to PSB attack in maize might be due to the strengthening of leaf cell wall components by increased hemicelluloses cross-linking and may also be due to a wider array of resistance mechanisms^12^. The differential response of maize genotypes may be due to induced and constitutive defenses, interaction compatibility in terms of maize susceptibility, PSB virulence and duration of its feeding. The occurrence of induced responses in the short-term would be of great advantage to maize which may reduce the subsequent herbivore attack^13^. The chewing insect pests induces the production of phytohormone, jasmonic acid which activates physiological responses resulting in the production of secondary metabolites and enhanced resistance^14^ in host plants. Herbivores also release salivary components while feeding on host-plants which results in the activation of defense mechanisms^15,16^. For example, in maize, leaf-feeding by Mediterranean corn borer (*Sesamia nonagroides*) and larval regurgitant played a positive role in eliciting defense response^12^.

The current study also examined how maize in response to mechanical wounding elicits defense responses. The mechanical wounding on V3 stage plants did not change *p*-CA content significantly in leaves of genotypes either in short- or in long-term. Probably the majority of *p*-CA content is already present constitutively and may not be induced by wounding at V3 stage. On the contrary this may also indicates that plants can distinguish damage from herbivore attack and the wounding due to the induction of different transcript profiles^17^. However, at V6 stage, CM 500 accumulated higher *p*-CA content (0.70 mg/g) in its leaves in short-term (Fp < 0.0001, LSD = 0.12) as compared to control (0.47 mg/g). Similarly, a higher accumulation of defense-related secondary metabolites were also observed in rice in response to attack from various herbivores such as *Spodoptera mauritia*, *Mythimna loreyi*, and *Parnara guttata* as compared to wounding alone^18,19^. Thus, mechanical wounding alone does not appear to play a significant role in eliciting defense responses in maize as compared to PSB feeding.

In the combination of wounding and regurgitation treatment, the differences among genotypes with respect to *p*-CA content in leaves at V3 stage were not significant in short-term response. However, WNZ ExoticPool (0.82 mg/g) accumulated significantly higher *p*-CA content in long-term response (Fp < 0.0001, LSD = 0.06). The *p*-CA content of leaves increased significantly at V6 stage in CM 500 (1.01 mg/g) in long-term response (Fp < 0.0001, LSD = 0.05). Earlier studies have shown that the defense-related genes and jasmonic acid would get induced in maize by the regurgitates from *Spodoptera exigua*, *Spodoptera littoralis* and *Mythimna separata*^20,21^. However, the direct defenses were not elicited due to the regurgitant of *Ostrinia nubilalis* in maize.

The exogenous application of MeJA onV3 stage plants did not alter *p-*CA content of leaves in short-term in any of the genotypes. However, in long-term, significant increase of *p*-CA content was observed in leaf tissues of CM 500 (0.74 mg/g) (Fp < 0.0001, LSD = 0.16) at V3 stage. Whereas exogenous application of MeJA onV6 stage plants led to higher accumulation of *p*-CA in leaves of DMRE 63 (0.72 mg/g) in short-term (Fp < 0.0001, LSD = 0.03) and long-term (0.82 mg/g) but in WNZ ExoticPool (0.78 mg/g) (Fp < 0.0001, LSD = 0.05) only long-term response was observed. The present finding is in agreement with some other researchers who also observed that exogenous application of methyl jasmonate induces plant defense mechanisms^22^. A cascade of events gets activated inside the plant tissues which results in the production of secondary defense metabolites due to the application of methyl jasmonate^23^.

Among all the treatments, PSB feeding alone showed significantly higher *p-*CA content in leaf tissues at V3 stage in both short-and long-term (Fp < 0.0001, LSD = 0.04) responses. The interaction effect between treatments and genotypes were found significant in both short-(Fp < 0.0001, F value = 82.02) and long-term responses (Fp < 0.0001, F value = 37.46). Similarly at V6 stage also PSB feeding alone showed significantly higher accumulation of *p-*CA in leaf tissues in short-term (Fp < 0.0001, LSD = 0.09) response. However, in long-term response at V6 stage, PSB feeding followed by a combination of wounding and regurgitation showed significantly higher accumulation of *p*-CA content (Fp < 0.0001, LSD = 0.02).The interaction effect between treatments and genotypes were found significant in short-(Fp < 0.0001, F value = 7.10) and long-term (Fp < 0.0001, F value = 229.37) responses indicated the induced defense responses depend on the type of genotype and treatment.

#### Changes in p-CA content after imposition of different treatments in stalk tissues

**Figure 3:**
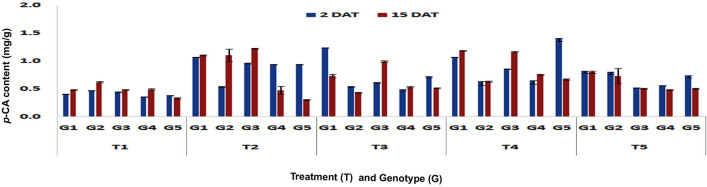
Changes in *p*-CA content in stalk tissues of maize genotypes to induced treatments in short (2 DAT) and long (15 DAT) term responses on the imposition of treatments at V3 stage (G1: DMRE 63; G2: CM 500; G3: WNZ Exoticpool; G4: CM 202; G5: BML 6; T_1_: Control; T_2_: PSB feeding; T_3_: Wounding; T_4_: Combination of wounding plus regurgitation; T_5_: Exposure to methyl jasmonate).

**Figure 4:**
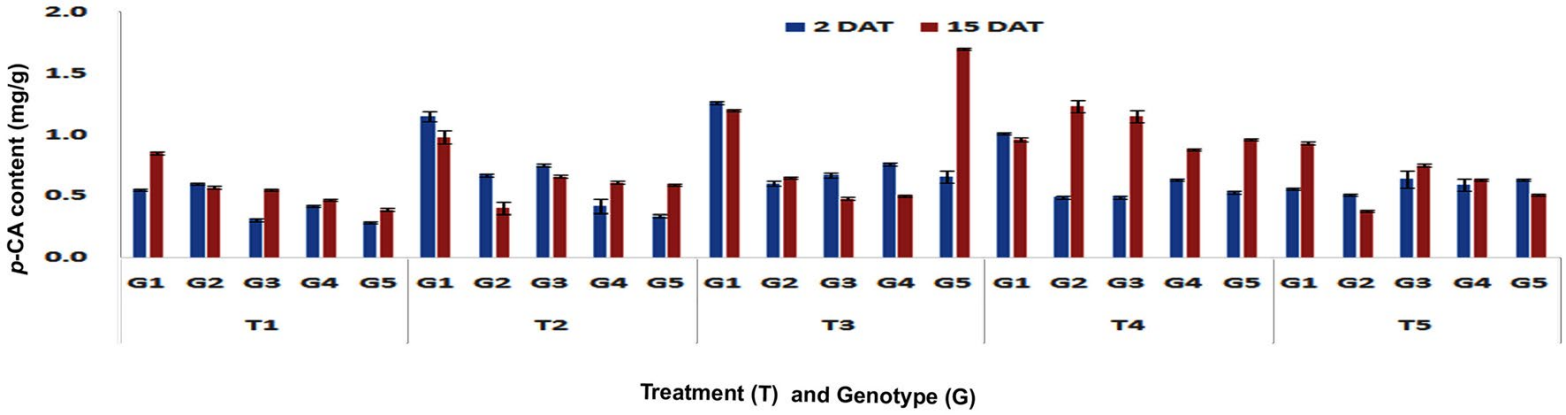
Changes in *p*-CA content in stalk tissues of maize genotypes to induced treatments in short (2 DAT) and long (15 DAT) term responses on the imposition of treatments at V6 stage (G1: DMRE 63; G2: CM 500; G3: WNZ Exoticpool; G4: CM 202; G5: BML 6; T_1_: Control; T_2_: PSB feeding; T_3_: Wounding; T_4_: Combination of wounding plus regurgitation; T_5_: Exposure to methyl jasmonate).

Significant higher *p-*CA content was accumulated in stalk tissues also upon feeding by PSB larvae on V3 stage plants of DMRE 63 (1.07 and 1.1 mg/g), CM 500 (0.54 and 1.1 mg/g) and WNZ ExoticPool (0.96 and 1.22 mg/g) in both short- (Fp < 0.0001, LSD = 0.01) and long-term (Fp < 0.0001, LSD = 0.07) responses, respectively (Fig. 3). Similar and significantly increasing trend of *p*-CA content was also observed in stalk tissues of DMRE 63 (1.15 and 0.98 mg/g) and WNZ ExoticPool (0.75 and 0.66 mg/g) at V6 stage also in both short- (Fp < 0.0001, LSD = 0.10) and long-term responses (Fp < 0.0001, LSD = 0.04), respectively (Fig. 4).

In contrast to leaves, considerable increase of *p*-CA content was found in stalk tissues due to mechanical wounding on V3 stage plants. DMRE 63 (1.24 and 0.73 mg/g) and WNZ ExoticPool (0.62 and 0.99 mg/g) have shown significantly higher *p*-CA content in short-(Fp < 0.0001, LSD = 0.02) and long-term (Fp < 0.0001, LSD = 0.02) responses, respectively. Similarly, the significant increase of *p*-CA content of stalk tissue at V6 stage was found in short-term (1.26 mg/g, Fp < 0.0001, LSD = 0.02) and long-term (1.2 mg/g, Fp < 0.0001, LSD = 0.01) in DMRE 63 and only long-term in WNZ ExoticPool (0.67 mg/g). Similarly, the significant increase in *p-*CA content of stalk was also observed in one of the susceptible genotype, BML6 in both short (0.66 mg/g) (Fp < 0.0001, LSD = 0.02) and long-term responses (1.70 mg/g) (Fp < 0.0001, LSD = 0.01). This is contrary to the predicted result as susceptible genotypes are expected to be a lesser accumulation of phenolic acids.

In the combination of wounding and regurgitation treatment, the content of *p*-CA in stalk tissues was significantly higher at V3 stage in DMRE 63 (1.07 and 1.18 mg/g) and WNZ ExoticPool (0.86 and 1.16 mg/g) in short- (Fp < 0.0001, LSD = 0.07) and long-term responses, (Fp < 0.0001, LSD = 0.01) respectively, whereas in CM 500 (0.63 mg/g) it was significantly higher in short-term response. The *p*-CA content of stalk in the susceptible genotype, BML 6 at V3 stage was also higher (1.41 mg/g) in short-term response. DMRE 63 accumulated significantly higher content of *p*-CA (1.01 mg/g) at V6 stage in short-term (Fp < 0.0001, LSD = 0.01) response whereas in long-term (Fp < 0.0001, LSD = 0.06) response, CM 500 (1.23 mg/g) and WNZ ExoticPool (1.15 mg/g) accumulated significantly more content of *p*-CA.

The exogenous application of MeJA on V3 stage plants has showed significantly higher accumulation of *p*-CA in the stalk tissues of DMRE 63 (0.82 mg/g) and CM 500 (0.80 mg/g) in short-term (Fp < 0.0001, LSD = 0.06). Simlarly both DMRE 63 (0.80 mg/g) and CM 500 (0.73 mg/g) also showed significantly higher *p*-CA content in long-term (Fp < 0.0001, LSD = 0.07) as well. Whereas at V6 stage, the stalk tissues in WNZ Exotic Pool have shown significantly higher *p*-CA content i.e. 0.64 mg/g and 0.75 mg/g in both short- (Fp < 0.0001, LSD = 0.10) and long-term (Fp < 0.0001, LSD = 0.01) responses, respectively. However DMRE 63 (0.93 mg/g) exhibited only in long-term response (Fp < 0.0001, LSD = 0.01). Similarly, the stalk tissues of BML6 also showed increased accumulation of *p*-CA in both short- and long-term responses but the increased content was much lesser than the resistant genotypes.

The combination of wounding and regurgitation followed by PSB feeding recorded significantly higher *p*-CA content in stalk tissues at V3 stage in both short- (Fp < 0.0001, LSD = 0.02) and long-term (Fp < 0.0001, LSD = 0.03) responses. The interaction effect between different treatments and genotypes were found significant in short-(Fp < 0.0001, F value = 129.45) and long-term responses (Fp < 0.0001, F value = 72.18). At V6 stage, wounding followed by PSB feeding induced significantly higher *p*-CA content in short-term response (Fp < 0.0001, LSD = 0.03), while in long-term response, wounding and regurgitation followed by wounding and PSB feeding induced higher *p*-CA content. The interaction effects between treatments and genotypes were found significant in short-(Fp < 0.0001, F value = 28.64) and long-term responses (Fp < 0.0001, F value = 282.3).The present study provided insight into how plants were able to recognize specifically the induced treatment and responded accordingly. However, further exploration is required to find out the exact mechanisms responsible for induced defences. In all the experiments that were conducted, treatment-specific and genotype-dependent changes were observed in the accumulation of *p*-CA content in both short- and long-term responses at V3 and V6 stages.

#### Changes in FA content after imposition of different treatments in leaf tissues

**Figure 5:**
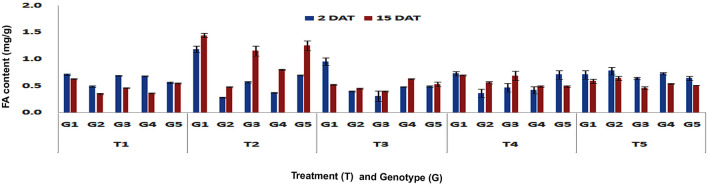
Changes in FA content in leaf tissues of maize genotypes to induced treatments in short (2 DAT) and long (15 DAT) term responses on the imposition of treatments at V3 stage (G1: DMRE 63; G2: CM 500; G3: WNZ Exoticpool; G4: CM 202; G5: BML 6; T_1_: Control; T_2_: PSB feeding; T_3_: Wounding; T_4_: Combination of wounding plus regurgitation; T_5_: Exposure to methyl jasmonate).

**Figure 6:**
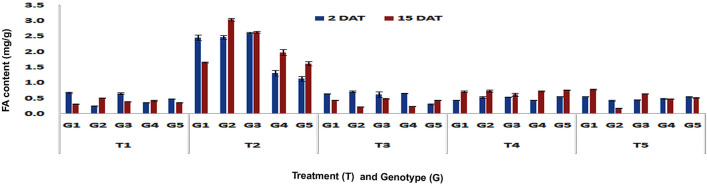
Changes in FA content in leaf tissues of maize genotypes to induced treatments in short (2 DAT) and long (15 DAT) term responses on the imposition of treatments at V6 stage (G1: DMRE 63; G2: CM 500; G3: WNZ Exoticpool; G4: CM 202; G5: BML 6; T_1_: Control; T_2_: PSB feeding; T_3_: Wounding; T_4_: Combination of wounding plus regurgitation; T_5_: Exposure to methyl jasmonate).

A significant increase in the FA content in resistant maize genotypes was observed upon different treatments in comparison to control. The leaf-feeding by pink stem borer larvae resulted in significant increase in FA content in leaves of DMRE 63 (1.18 and 1.44 mg/g) at V3 stage as compared to control (0.71 and 0.63 mg/g), in both short- (Fp < 0.0001, LSD = 0.15) and long-term (Fp < 0.0001, LSD = 0.14) responses, respectively (Fig. 5). However, WNZ ExoticPool accumulated significantly higher FA content (1.15 mg/g) only in long-term response. The BML 6, a susceptible genotype showed increased accumulation of FA content (1.25 mg/g) in leaves at V3 stage in long-term response but it was less than the resistant genotype DMRE 63. At V6 stage, the leaf-feeding by PSB larvae resulted in significant increase of FA content in the leaves of DMRE 63 (2.46 and 1.66 mg/g), CM 500 (2.47 and 3.04 mg/g) and WNZ ExoticPool (2.61 and 2.64 mg/g) as compared to control treatments of DMRE 63 (0.68 and 0.32 mg/g), CM 500 (0.25 and 0.51 mg/g) and WNZ ExoticPool (0.65 and 0.39 mg/g) in both short- (Fp < 0.0001, LSD = 0.66) and long-term (Fp < 0.0001, LSD = 0.07) responses, respectively (Fig. 6). Interestingly, the increased FA content in leaves infested by PSB was also observed in susceptible genotypes CM 202 (1.31 and 1.98 mg/g) and BML 6 (1.13 and 1.62 mg/g) as compared to untreated control. However, the increased content in leaf and stalk tissues upon PSB infestation was significantly lower than the resistant and moderately resistant genotypes.

Mechanical wounding at V3 stage increased the FA content significantly in leaves of DMRE 63 (0.95 mg/g) in short-term (Fp < 0.0001, LSD = 0.03) response, whereas in CM 500 (0.45 mg/g) and CM 202 (0.63 mg/g) it was observed in long-term responses. Similarly mechanical wounding at V6 stage resulted in slight increase of FA content in leaves of CM 500 (0.71 mg/g) in short-term (Fp < 0.0001, LSD = 0.12), while DMRE 63 (0.44 mg/g) and WNZ ExoticPool (0.49 mg/g) exhibited in long-term response.

The mechanical wounding followed by application of regurgitation on V3 stage plants did not show any significant differences in the FA content of leaves among different genotypes in short-term response as compared to control. However, in long-term response significant increase (Fp < 0.0001, LSD = 0.05) in FA content was observed in the leaves of DMRE 63 (0.7 mg/g), CM 500 (0.56 mg/g) and WNZ ExoticPool (0.69 mg/g) at V3 stage. Whereas at V6 stage, only CM 500 accumulated considerable higher FA content (0.54 mg/g) in its leaf tissues in short-term responses as compared to control (0.25 mg/g). Interestingly, in the long-term response, all the genotypes accumulated higher FA as compared to control.

The exogenous application of MeJA resulted in increased content of FA in leaves of CM 500 (0.78, 0.64 mg/g) at V3 stage in short- (Fp < 0.0001, LSD = 0.07) and long-term (Fp < 0.0001, LSD = 0.16) responses. Whereas at V6 stage, the increase in concentration of FA was significant in leaves of only CM 500 (0.43 mg/g) in short-term response, however it was observed in DMRE 63 (0.79 mg/g) and WNZ ExoticPool (0.65 mg/g) in long-term response.

It was observed that maize genotypes respond differently to insect interaction i.e. PSB feeding and non-insect interactions namely mechanical wounding, a combination of wounding and regurgitation, and exogenous application of methyl jasmonate when compared to control.

The FA content in leaves at V3 stage did not show any significant differences among different induced treatments in the short-term while in long-term, PSB feeding followed by a combination of wounding and regurgitation and exogenous application of MeJA showed significant differences (Fp < 0.0001, LSD = 0.04) as compared to control. The interaction effect between treatments and genotypes were found significant in short- (Fp < 0.0001, F value = 9.72) and long- term responses (Fp < 0.0001, F value = 25.37). Whereas at V6 stage, only PSB feeding showed significantly higher FA content in leaf tissues in short- (Fp < 0.0001, LSD = 0.18) and long-term responses (Fp < 0.0001, LSD = 0.02). The interaction effect between treatments and genotypes were found significant in short- (Fp < 0.0001, F value = 4.97) and long-term (Fp < 0.0001, F value = 82.02) responses.

#### Changes in FA content after imposition of different treatments in stalk tissues

**Figure 7:**
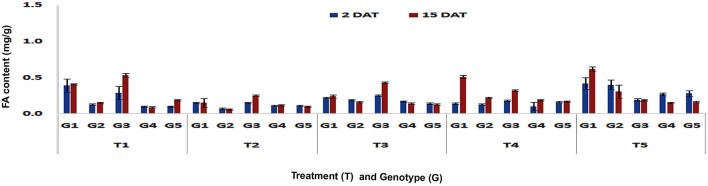
Changes in FA content in stalk tissues of maize genotypes to induced treatments in short (2 DAT) and long (15 DAT) term responses on the imposition of treatments at V3 stage (G1: DMRE 63; G2: CM 500; G3: WNZ Exoticpool; G4: CM 202; G5: BML 6; T_1_: Control; T_2_: PSB feeding; T_3_: Wounding; T_4_: Combination of wounding plus regurgitation; T_5_: Exposure to methyl jasmonate).

**Figure 8:**
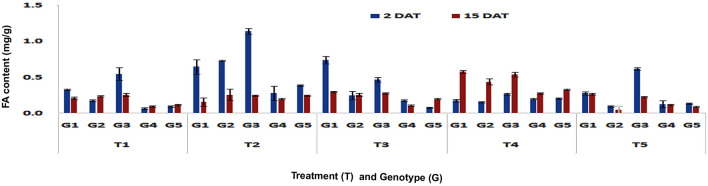
Changes in FA content in stalk tissues of maize genotypes to induced treatments in short (2 DAT) and long (15 DAT) term responses on the imposition of treatments at V6 stage (G1: DMRE 63; G2: CM 500; G3: WNZ Exoticpool; G4: CM 202; G5: BML 6; T_1_: Control; T_2_: PSB feeding; T_3_: Wounding; T_4_: Combination of wounding plus regurgitation; T_5_: Exposure to methyl jasmonate).

The FA content did not increase in stalk tissues upon PSB feeding in any of the genotypes at V3 stage either in short-or in long-term (Fig. 7). However at V6 stage, significantly higher concentrations of FA was observed in DMRE 63 (0.65 mg/g), CM 500 (0.73 mg/g) and WNZ Exotic Pool (1.14 mg/g) in short-term response (Fp < 0.0001, LSD = 0.08) (Fig. 8). On the contrary, no significant increase in FA content was observed in any of the genotypes in the long-term response. The FA content in stalk tissue after mechanical wounding at V3 stage did not show any differences among the genotypes in both short- and long-term responses. Whereas mechanical wounding at V6 stage exhibited increased content of FA in DMRE 63 (0.74 mg/g) in short term (Fp < 0.0001, LSD = 0.07) response as compared to control treatment. The combination of mechanical wounding followed by application of regurgitation treatment at V3 and V6 stages did not show any significant differences in accumulation of FA content of stalk in short-term response. However, genotypes DMRE 63 and CM 500 exhibited significantly higher (Fp < 0.0001, LSD = 0.02) accumulation of FA content 0.51 and 0.22 mg/g respectively in long-term response. Whereas at V6 stage, the FA content was increased significantly in stalk tissues of DMRE 63 (0.58 mg/g), WNZ ExoticPool (0.54 mg/g) and CM 500 (0.44 mg/g) in long-term (Fp < 0.0001, LSD = 0.04) response as compared to control. The FA content in stalk after exogenous application of MeJA at V3 stage increased significantly in CM 500 (0.4 mg/g) in short-term (Fp < 0.0001, LSD = 0.03) response; while in long-term, DMRE 63 (0.62 mg/g) and CM 500 (0.31 mg/g) showed significant increase in FA content (Fp < 0.0001, LSD = 0.09). The concentration of FA in stalk tissues at V6 stage also increased significantly in WNZ Exotic Pool (0.62 mg/g) and DMRE 63 (0.27 mg/g) in short- (Fp < 0.0001, LSD = 0.06) and long-term responses (Fp < 0.0001, LSD = 0.02), respectively. In the case of stalk tissues among all treatments, the exogenous application of MeJA at V3 stage showed significant increase in FA content in short-term response. The interaction effect between treatments and genotypes were found significant in short-term (Fp < 0.0001, F value = 82.02) and long-term (Fp < 0.0001, F value = 12.08) responses. Whereas at V6 stage, PSB feeding exhibited significantly higher content of FA in stalk tissues in short-term response (Fp < 0.0001, LSD = 0.03), while in long-term response, the combination of wounding and regurgitation followed by PSB feeding led to higher FA content accumulation (Fp < 0.0001, LSD = 0.02). The result suggested that contact of regurgitant elicitors with the maize plant may be responsible for increased content of FA in treated plants. Furthermore, the interaction effect between treatments and genotypes were found significant in short- (Fp < 0.0001, F value = 24.74) and long-term responses (Fp < 0.0001, F value = 16.98).

It was implied that the efficiency of the induced response depends on its magnitude and spatial extent^24^. The present study coordinates the potential interactions of phenolic acids responsible for plant resistance at different stages of crop growth. The increased levels of *p-*CA and FA content direct their role in plant defense by inducing the signaling pathways. However, these findings require further exploration in identifying different mechanisms of induced resistance. Methyl Jasmonate acts as a signaling molecule in maize defense against chewing insects^25^ such as Mediterranean corn borer^26^ and Asian corn borer^27^. However, the ethylene-mediated response is involved in *Arabidopsis* against Egyptian cotton worm^28^. It was stated that these signaling pathways lead to changes in the expression of defense-related genes^29^. The results obtained in the present study might be due to identification of the defense elicitors by plants from herbivores by activating kinase networks and phytohormones. It was also reported that cross-talks occur between signaling pathways about the induction of specific responses by insect attack^30^. However, the mechanisms underlying these regulations still need to be explored. Similarly, induced resistance was observed in the undamaged parts of the same plant and also in the neighbouring plants^31^. In wild radish, *Raphanus raphanistrum,* transgenerational effect, i.e. transferring from parents to their offspring was reported in response to *Pieris rapae* infestation^32^. This strategy of transgenerational induced resistance makes plants more vigorous and reduces insect infestation in progeny. It was opined that plants have to maintain the allocation of resources to growth and defense responses and thus inducible defenses must be precisely regulated^33^. This in-turn exhibits specific plant responses to varied herbivore challenges.

## Conclusion

The findings in the present study provided important evidence that insect interaction-PSB feeding, and non-insect interactions including a combination of wounding and regurgitation, mechanical wounding, and exposure to methyl Jasmonate may induce defense responses in maize. However, PSB feeding strongly induced defense responses resulted in the accumulation of higher content of phenolic acids-*p*-CA and FA in leaf tissues of resistant and moderately resistant genotypes possibly contributing to enhanced resistance in maize. In stalk tissues, defense responses were induced due to wounding and regurgitation followed by PSB feeding. It should be noted that in the present study, induced defense responses varied with maize genotypes, stage of crop growth, plant tissue, in terms of both short- and long-term responses. The results obtained are useful for future breeding programs aiming at resistance to PSB infestation. Further, substantial yield losses and insecticidal applications can be minimized resulting in an eco-friendly environmental footprint. However, the in-depth studies on underlying mechanisms of induced defense responses of maize have yet to be explored for effective and sustainable PSB management.

## Methods

### Insect rearing

The PSB larvae were obtained from the colony maintained at the Winter Nursery Centre, ICAR-Indian Institute of Maize Research (ICAR-IIMR), Hyderabad, Telangana, India^34^.

### Plant materials

The experimental material was comprised of five maize genotypes which included resistant (DMRE63 and CM 500), moderately resistant (WNZ ExoticPool), and susceptible (CM 202 and BML 6) genotypes to PSB (Table 1). The above genotypes with varying degrees of resistance and susceptibility to PSB were selected based on 3 years of screening^35,36^.

### Experimental design and treatment applications

The experiment was conducted in two different sets one each to record the response of genotypes upon PSB infestation and defense responses of the genotypes against different treatments in field and pot, respectively. The field experiment was conducted in a plot size of 7.5 m^2^ during *Rabi* 2019–20 by following a randomized complete block design (RCBD) with four replications at Winter Nursery Centre (17.3254 N; 78.4004 E; 527 amsl), ICAR-IIMR, Rajendranagar, Hyderabad, Telangana, India. The experimental unit or plot comprised of four rows of 2.5 m length with 75 × 20 cm spacing between rows and plants within a row, respectively. The crop was raised by following recommended agronomic practices for inbred lines. The total number of plants per plot maintained was 48 @ 12/row before infestation. At 12 days after germination (DAG), second-generation neonate larvae of PSB reared from field population were released into the whorls of plants @ 10 larvae/plant with the help of camel hair brush^37^. The visual rating of leaf injury rating (LIR) was recorded on a 1–9 scale at 35 days after infestation^38^. The resistant, moderately resistant, and susceptible lines were defined by LIR 1–3, > 3.1–6 and > 6.1–9, respectively. (1) Plant with absolutely no damage; (2) Plant with pin sized holes of size 2–3 mm on few leaves; (3) Plant with elongated holes of size 5 mm on few leaves; (4) Plant with injury in about 1/3 of total number of leaves and midrib damage on 1–2 leaves; (5) Plant with about 50% leaf damage, slits and streaks of 5–10 cms and midrib damage on leaves; (6) Plant with leaf injuries to about two thirds of the total number of leaves(ragged appearance) or slits at the base of the stem with > 10 cms streaks; (7) Plant with every type of leaf injury and almost all the leaves damaged with tassel stalk boring or circular dark ring at the base of stem; (8) Plant with stunted growth in which the entire foliage is damaged; (9) Plant with dried central whorl (Dead Heart). The description of visual rating of LIR has been mentioned based on the standard reference^38^.

To investigate the maize defense responses against different treatments, the second experiment was conducted by growing maize plants individually in pots. Five treatments were designed to study induced defense responses including insect and non-insect interactions namely (i) control (untreated plants) (T_1_) (ii) PSB larvae infestation (T_2_), (iii) mechanical wounding (T_3_), (iv) mechanical wounding plus PSB regurgitation (T_4_), (v) exposure to methyl jasmonate (T_5_). The treatments were imposed at two stages i.e. V3 and V6 stages. The pots were kept in a completely randomized design involving a 5 × 5 factorial arrangement with five treatments, five genotypes at two levels of sampling. In the control treatment (T_1_), plants were not exposed to any treatment. In the T_2_ treatment, three 3rd instar larvae were released into the whorl of the maize plants and were allowed to feed freely. The pots were protected with cage-nets to avoid larval dispersion. In the third treatment (T_3_), three-leaf wounds of 6 × 6 mm were made with a scalpel on each plant and 5 μl phosphate buffer solution (PBS) (15 μl PBS per plant) was applied to each wound. In the treatment T_4_, the PSB regurgitant was collected from 4th instar larvae previously fed on maize for at least 48 h. For regurgitant collection, larvae were chilled on ice and immobilized. After returning to room temperature, the larvae were squeezed until the regurgitant was expelled. Approximately 300 μl regurgitant collected from 50 larvae was mixed with half-volume i.e. 150 μl 0.1 M PBS and the mixture was frozen. Six micro-liter regurgitant mixture was applied to plants immediately after wounding using a micropipette. The total amount of regurgitant mixture exposed to each plant was 18 μl (containing regurgitant from approximately 3–4 larvae). In the T_5_ treatment, plants were exposed to exogenous MeJA, by placing cotton tip soaked with 100 μMMeJA dissolved in ethanol (10% v/v) in a leaf axil and also between stalks. The leaf and stalk samples were collected from different plants of each treatment–genotype combination at two and 15 days after treatment (DAT) for quantification of cell wall phenolic monomers. The experimental replicate consisted of pooled material from at least five leaves from each of three plants.

### Extraction of p-CA and FA

The leaf and stalk samples were crushed in 80 percent chilled ethanol (1:10 w/v) to extract phenolic acids. A sample of 1000 mg of grounded leaf and stalk tissues were used for cell wall monomers extraction. After extraction, the contents were shaken in a rotary water-bath for 20 min at room temperature. The samples were centrifuged at 5000 rpm for 5 min and the resulting pellets containing bound phenolic acids were stored at -40 °C until alkaline hydrolysis^39^. Since some soluble phenols can be present in the pellets, the pellet/residue was re-extracted twice with 10 ml of 80% chilled ethanol before proceeding for alkaline hydrolysis. The pellet was taken into a tube and 10 ml of 4 N sodium hydroxide was added and the mixture was flushed with nitrogen gas for 30 s to create an inert environment. The flask containing an alkaline hydrolyzed mixture was sealed and kept for shaking on a rotary water bath at 65 °C for 90 min. After alkaline hydrolysis, the mixture was acidified to pH 2 with 6 N HCl. Subsequently, the mixture was centrifuged at 5000 rpm for 5 min, to remove the flocculated material. The acidified bound phenolic acids were extracted in n-hexane (1:1 v/v) five times and the upper layer was discarded. The residue was extracted with ethyl acetate six times. Ethyl acetate fractions were combined, evaporated to dryness under a water bath, and was reconstituted in 5 ml of 70 percent methanol, and stored at − 40 °C for analysis.

### Determination of p-CA and FA

The *p*-CA and FA content were determined using Shimadzu Ultra-Fast Liquid Chromatography (UFLC) equipped with an SPD-M20A Prominence photodiode array detector. The HPLC pumps, autosampler, column temperature, and diode array system were monitored and controlled using the LC Solution Chromatography data software program. *p*-CA and FA separation were performed on the C18-Phenomenex column (250 × 4.6 mm). The column was held at 35 °C and the flow rate was set at 1.0 ml per minute. The solvent system consisted of 2 percent glacial acetic acid (A) and 100 percent Acetonitrile (B). A gradient program of 15 percent B and 85 percent A for 20 min was followed with a 20 µl sample injection. The analysis was done by preparing samples in three replications. The peaks of *p*-CA and FA were identified by standards at 280 nm with retention time 10.9 and 11.6 min, respectively. The amount of *p*-CA and FA in maize leaf and stalk samples were quantified by the calibration curve of respective standards with the help of LC Solution Software (Supplementary Information).

### Statistical analysis

The effect of induced treatments on *p*-CA and FA content at each sampling time were subjected to two-way ANOVA by using a general linear model (PROC GLM). The one-way ANOVA was also performed to know the significant differences among genotypes in each induced treatment compared to untreated control. The data for leaf injury rating (LIR) was generated in randomized complete block design and analysis was performed by using PROC ANOVA. The significant differences among the genotype means were judged by using the least significant differences (LSD). All the analyses were performed in SAS version 9.3^40^.

## Supplementary Information

Supplementary Tables.
