# Supplementary material for: Induction of cell wall phenolic monomers as part of direct defense response in maize to pink stem borer (Sesamia inferens Walker) and non-insect interactions
Source: Sci Rep. 2021 Jul 20;11:14770. doi: 10.1038/s41598-021-93727-2 (PMC8292465; doi:10.1038/s41598-021-93727-2)
Supplement: Supplementary file 1 — Supplementary Tables. [file 41598_2021_93727_MOESM1_ESM.docx]

**Supplementary Table 1. Changes in *p*-CA content in leaf tissues of maize genotypes to induced treatments in short (2 DAT) and long (15 DAT) term responses on the imposition of treatments atV3 and V6 phenological stages**

|  | **V3 Stage** | | **V6 Stage** | |
| --- | --- | --- | --- | --- |
|  | **2 DAT** | **15 DAT** | **2 DAT** | **15 DAT** |
| **Untreated Control** |  |  |  |  |
| DMRE 63 | 1.19±0.01^bc^ | 1.07±0.01^c^ | 1.08±0.03^bc^ | 0.72±0.01^j^ |
| CM 500 | 0.79±0.01^efg^ | 0.63±0.03 ^ghijk^ | 0.47±0.02 ^j^ | 0.72±0.01^ij^ |
| WNZ ExoticPool | 1.10±0.01^c^ | 0.67±0.02^gh^ | 0.87±0.03^cdef^ | 0.70±0.02 ^j^ |
| CM 202 | 1.19±0.06^bc^ | 0.60±0.02 ^hijkl^ | 0.61±0.01^fghij^ | 0.70±0.02 ^j^ |
| BML 6 | 0.89±0.01^de^ | 0.87±0.02^d^ | 0.73±0.01^defgh^ | 0.63±0.01 ^k^ |
| **PSB feeding** |  |  |  |  |
| DMRE 63 | 1.24±0.02^b^ | 1.67±0.04^a^ | 1.26±0.09^b^ | 1.03±0.01^d^ |
| CM 500 | 0.44±0.01^l^ | 0.66±0.01^ghij^ | 1.28±0.04^b^ | 1.94±0.03^a^ |
| WNZ ExoticPool | 2.23±0.01^a^ | 1.48±0.06 ^b^ | 1.66±0.01 ^a^ | 1.60±0.02^c^ |
| CM 202 | 0.54±0.03^kl^ | 0.69±0.01^gh^ | 1.19±0.05^b^ | 0.93±0.04^e^ |
| BML 6 | 1.10±0.08 ^c^ | 0.84±0.04^de^ | 0.72±0.04^defgh^ | 1.72±0.03^b^ |
| **Mechanical Wounding** |  |  |  |  |
| DMRE 63 | 0.89±0.05^de^ | 0.55±0.01^ijkl^ | 0.88±0.02 ^cde^ | 0.54±0.04 ^l^ |
| CM 500 | 0.80±0.05^ef^ | 0.61±0.01 ^ijkl^ | 0.70±0.02 ^efghi^ | 0.35±0.01^m^ |
| WNZ ExoticPool | 0.53±0.01^kl^ | 0.55±0.01^jkl^ | 0.73±0.02 ^defgh^ | 0.60±0.01^k^ |
| CM 202 | 0.52±0.01^kl^ | 0.71±0.01^fgh^ | 0.83±0.01 ^defg^ | 0.37±0.01^m^ |
| BML 6 | 0.60±0.01^jk^ | 0.66±0.06^ghi^ | 0.47±0.01^j^ | 0.64±0.01^k^ |
| **Wounding + Regurgitation** |  |  |  |  |
| DMRE 63 | 0.72±0.03 ^fghi^ | 0.87±0.01^d^ | 0.50±0.01^ij^ | 0.77±0.03^hi^ |
| CM 500 | 0.52±0.01^kl^ | 0.63±0.01^ghijk^ | 0.55±0.02^h^ | 1.01±0.05^d^ |
| WNZ ExoticPool | 0.69±0.01^ghij^ | 0.82±0.01^def^ | 0.60±0.01^hij^ | 0.88±0.06^g^ |
| CM 202 | 0.74±0.02^fgh^ | 0.55±0.01 ^jkl^ | 0.77±0.02^defgh^ | 0.88±0.01^f^ |
| BML 6 | 0.94±0.08^d^ | 0.61±0.06 ^hijkl^ | 0.65±0.01^cd^ | 0.85±0.01^gf^ |
| **Exposure to methyl jasmonate** |  |  |  |  |
| DMRE 63 | 0.62±0.04 ^ijk^ | 0.53±0.01^kl^ | 0.72±0.01^defgh^ | 0.82±0.01^g^ |
| CM 500 | 0.82±0.04 ^ef^ | 0.74±0.02^efg^ | 0.64±0.01^ghij^ | 0.29±0.01^n^ |
| WNZ ExoticPool | 0.67±0.01 ^hij^ | 0.50±0.08 ^l^ | 0.63±0.01^ghij^ | 0.78±0.01^h^ |
| CM 202 | 0.71±0.01^fghi^ | 0.67±0.01^gh^ | 0.66±0.01^ghij^ | 0.71±0.01 ^j^ |
| BML 6 | 0.76±0.04 ^fgh^ | 0.66±0.03^ghi^ | 0.62±0.01^ghij^ | 0.73±0.01 ^ij^ |

Each value represents the mean ± SEm of 3 replications. Means within a column followed by different letters are significantly different (LSDTest p =0.05)

**Supplementary Table 2. Changes in *p*-CA content in stalk tissues of maize genotypes to induced treatmentsin short (2 DAT) and long (15 DAT) term responses on the imposition of treatments atV3 and V6 phenological stages**

|  | **V3 Stage** | | **V6 Stage** | |
| --- | --- | --- | --- | --- |
|  | **2 DAT** | **15 DAT** | **2 DAT** | **15 DAT** |
| **Untreated Control** |  |  |  |  |
| DMRE 63 | 0.41±0.01^mn^ | 0.48±0.01^hi^ | 0.55±0.01^ghij^ | 0.85±0.01^f^ |
| CM 500 | 0.47±0.01^kl^ | 0.62±0.01^g^ | 0.60±0.01^fgh^ | 0.57±0.01^kl^ |
| WNZ ExoticPool | 0.45±0.02^lm^ | 0.48±0.01^hi^ | 0.31±0.01^m^ | 0.55±0.01^lm^ |
| CM 202 | 0.36±0.01^n^ | 0.49±0.01^hi^ | 0.42±0.01^kl^ | 0.47±0.01^n^ |
| BML 6 | 0.38±0.01^n^ | 0.33±0.01^j^ | 0.29±0.01^m^ | 0.39±0.01 ^o^ |
| **PSB feeding** |  |  |  |  |
| DMRE 63 | 1.07±0.01^c^ | 1.10±0.0 ^b^ | 1.15±0.04 ^b^ | 0.98±0.05^d^ |
| CM 500 | 0.54±0.01 ^i^ | 1.10±0.11^b^ | 0.67±0.01^def^ | 0.40±0.05^o^ |
| WNZ ExoticPool | 0.96±0.01^d^ | 1.22±0.01^a^ | 0.75±0.01^d^ | 0.66±0.01^h^ |
| CM 202 | 0.94±0.01^d^ | 0.47±0.07 ^hi^ | 0.42±0.06 ^kl^ | 0.61±0.01 ^ijk^ |
| BML 6 | 0.94±0.01^d^ | 0.3±0.01^j^ | 0.34±0.01^lm^ | 0.59±0.01^jkl^ |
| **Mechanical Wounding** |  |  |  |  |
| DMRE 63 | 1.24±0.01^b^ | 0.73±0.03^ef^ | 1.26±0.01^a^ | 1.20±0.01^bc^ |
| CM 500 | 0.54±0.01 ^ij^ | 0.43±0.01^i^ | 0.60±0.02^efghi^ | 0.65±0.01 ^hi^ |
| WNZ ExoticPool | 0.62±0.02^h^ | 0.99±0.01^c^ | 0.67±0.02^d^ | 0.48±0.01^n^ |
| CM 202 | 0.49±0.03 ^jkl^ | 0.53±0.01^h^ | 0.76±0.01^d^ | 0.50±0.01^n^ |
| BML 6 | 0.72±0.02^g^ | 0.51±0.01^h^ | 0.66±0.05^ef^ | 1.70±0.01^a^ |
| **Wounding + Regurgitation** |  |  |  |  |
| DMRE 63 | 1.07±0.01^c^ | 1.18±0.01^a^ | 1.01±0.01^c^ | 0.96±0.02^e^ |
| CM 500 | 0.63±0.07 ^h^ | 0.63±0.01^g^ | 0.49±0.01^k^ | 1.23±0.05^b^ |
| WNZ ExoticPool | 0.86±0.01^e^ | 1.16±0.01^ab^ | 0.49±0.01^jk^ | 1.15±0.05^c^ |
| CM 202 | 0.65±0.07^h^ | 0.75±0.01^de^ | 0.63±0.01^efg^ | 0.88±0.01^f^ |
| BML 6 | 1.41±0.06^a^ | 0.67±0.01^fg^ | 0.53±0.01^hij^ | 0.96±0.01^e^ |
| **Exposure to methyl jasmonate** |  |  |  |  |
| DMRE 63 | 0.82±0.01^ef^ | 0.80±0.02^d^ | 0.56±0.01^ghij^ | 0.93±0.01^e^ |
| CM 500 | 0.80±0.03^f^ | 0.73±0.02^ef^ | 0.51±0.01^ijk^ | 0.38±0.01^o^ |
| WNZ ExoticPool | 0.52±0.01^ijk^ | 0.50±0.01^hi^ | 0.64±0.07^efg^ | 0.75±0.01^g^ |
| CM 202 | 0.56±0.03^i^ | 0.48±0.01^hi^ | 0.59±0.05 ^fghi^ | 0.63±0.01^hij^ |
| BML 6 | 0.74±0.01^g^ | 0.50±0.01^hi^ | 0.63±0.01^efg^ | 0.51±0.01 ^mn^ |

Each value represents the mean ± SEm of 3 replications. Means within a column followed by different letters are significantly different (LSDTest p =0.05)

**Supplementary Table 3. Changes in FA content in leaf tissues of maize genotypes to induced treatmentsin short (2 DAT) and long (15 DAT) term responses on the imposition of treatments atV3 and V6 phenological stages**

|  | **V3 Stage** | | **V6 Stage** | |
| --- | --- | --- | --- | --- |
|  | **2 DAT** | **15 DAT** | **2 DAT** | **15 DAT** |
| **Untreated Control** |  |  |  |  |
| DMRE 63 | 0.71±0.01 ^cd^ | 0.63±0.01 ^def^ | 0.68±0.02 ^c^ | 0.32±0.01^o^ |
| CM 500 | 0.49±0.01 ^fgh^ | 0.35±0.01 ^k^ | 0.25±0.01 ^d^ | 0.51±0.01^j^ |
| WNZ ExoticPool | 0.69±0.01 ^cde^ | 0.46±0.01 ^ghijk^ | 0.65±0.03 ^cd^ | 0.39±0.01 ^mn^ |
| CM 202 | 0.68±0.01^cde^ | 0.36±0.01 ^jk^ | 0.36±0.01 ^cd^ | 0.43±0.01 ^lm^ |
| BML 6 | 0.56±0.01^efg^ | 0.55±0.01 ^efgh^ | 0.48±0.01 ^cd^ | 0.36±0.01^no^ |
| **PSB feeding** |  |  |  |  |
| DMRE 63 | 1.18±0.06^a^ | 1.44±0.04 ^a^ | 2.46±0.09 ^a^ | 1.66±0.02 ^d^ |
| CM 500 | 0.28±0.01^i^ | 0.48±0.01 ^ghijk^ | 2.47±0.06 ^a^ | 3.04±0.05 ^a^ |
| WNZ ExoticPool | 0.57±0.01^defg^ | 1.15±0.09 ^b^ | 2.61±0.02 ^a^ | 2.64±0.03 ^b^ |
| CM 202 | 0.37±0.01^hi^ | 0.80±0.01^c^ | 1.31±0.09 ^b^ | 1.98±0.10 ^c^ |
| BML 6 | 0.7±0.01^cde^ | 1.25±0.09 ^b^ | 1.13±0.08 ^b^ | 1.62±0.07 ^d^ |
| **Mechanical Wounding** |  |  |  |  |
| DMRE 63 | 0.95±0.07 ^b^ | 0.52±0.01 ^efghi^ | 0.65±0.01^cd^ | 0.44±0.01 ^kl^ |
| CM 500 | 0.40±0.01^hi^ | 0.45±0.01 ^hijk^ | 0.71±0.03 ^c^ | 0.23±0.01 ^p^ |
| WNZ ExoticPool | 0.31±0.01 ^i^ | 0.4±0.01 ^ijk^ | 0.63±0.09 ^cd^ | 0.49±0.01^jk^ |
| CM 202 | 0.48±0.01^gh^ | 0.63±0.01^de^ | 0.66±0.01^cd^ | 0.24±0.01^p^ |
| BML 6 | 0.49±0.01^fgh^ | 0.53±0.04 ^efghi^ | 0.31±0.01^cd^ | 0.44±0.01^e^ |
| **Wounding + Regurgitation** |  |  |  |  |
| DMRE 63 | 0.73±0.04 ^c^ | 0.70±0.01^cd^ | 0.44±0.01^cd^ | 0.72±0.03 ^gh^ |
| CM 500 | 0.36±0.08 ^hi^ | 0.56±0.02 ^defgh^ | 0.54±0.03 ^cd^ | 0.74±0.04 ^fg^ |
| WNZ ExoticPool | 0.47±0.08 ^gh^ | 0.69±0.09 ^cd^ | 0.54±0.01 ^cd^ | 0.62±0.05 ^i^ |
| CM 202 | 0.42±0.07 ^ghi^ | 0.49±0.01 ^ghijk^ | 0.44±0.01 ^cd^ | 0.73±0.01^g^ |
| BML 6 | 0.71±0.08 ^cd^ | 0.49±0.01 ^fghij^ | 0.56±0.01 ^cd^ | 0.77±0.01^hi^ |
| **Exposure to methyl jasmonate** |  |  |  |  |
| DMRE 63 | 0.71±0.08 ^cd^ | 0.59±0.04 ^defg^ | 0.56±0.01 ^cd^ | 0.79±0.01 ^f^ |
| CM 500 | 0.78±0.07 ^c^ | 0.64±0.04 ^de^ | 0.43±0.01 ^cd^ | 0.18±0.01^p^ |
| WNZ ExoticPool | 0.64±0.02 ^cdef^ | 0.46±0.03 ^ghijk^ | 0.45±0.01 ^cd^ | 0.65±0.01 ^i^ |
| CM 202 | 0.73±0.02 ^c^ | 0.54±0.01^defgh^ | 0.49±0.01 ^cd^ | 0.48±0.01^jkl^ |
| BML 6 | 0.64±0.04 ^cdef^ | 0.50±0.01 ^efghij^ | 0.55±0.01 ^cd^ | 0.52±0.01^j^ |

Each value represents the mean ± SEm of 3 replications. Means within a column followed by different letters are significantly different (LSDTest p =0.05)

**Supplementary Table 4. Changes in FA content in stalk tissues of maize genotypes to induced treatmentsin short (2 DAT) and long (15 DAT) term responses on the imposition of treatments atV3 and V6 phenological stages**

|  | **V3 Stage** | | **V6 Stage** | |
| --- | --- | --- | --- | --- |
|  | **2 DAT** | **15 DAT** | **2 DAT** | **15 DAT** |
| **Untreated Control** |  |  |  |  |
| DMRE 63 | 0.39±0.09 ^a^ | 0.41±0.01^c^ | 0.33±0.01^gh^ | 0.21±0.02 ^ghi^ |
| CM 500 | 0.13±0.01 ^fghi^ | 0.15±0.01 ^hijkl^ | 0.18±0.01 ^jklm^ | 0.24±0.01 ^efgh^ |
| WNZ ExoticPool | 0.29±0.01 ^b^ | 0.53±0.03 ^b^ | 0.55±0.09 ^de^ | 0.26±0.02 ^def^ |
| CM 202 | 0.10±0.01^ij^ | 0.09±0.01 ^lm^ | 0.07±0.01^o^ | 0.10±0.01^k^ |
| BML 6 | 0.10±0.01 ^ij^ | 0.19±0.01 ^fghi^ | 0.10±0.01^mno^ | 0.12±0.01^jk^ |
| **PSB feeding** |  |  |  |  |
| DMRE 63 | 0.15±0.01^efghi^ | 0.15±0.06 ^hijkl^ | 0.65±0.1^bc^ | 0.16±0.06 ^ij^ |
| CM 500 | 0.07±0.0 ^j^ | 0.06±0.01^m^ | 0.73±0.01^b^ | 0.26±0.08 ^def^ |
| WNZ ExoticPool | 0.15±0.01^efghi^ | 0.25±0.01^ef^ | 1.14±0.04 ^a^ | 0.25±0.01^efg^ |
| CM 202 | 0.11±0.01^hij^ | 0.12±0.01^jklm^ | 0.28±0.1^hi^ | 0.20±0.01^hi^ |
| BML 6 | 0.11±0.01^ghij^ | 0.10±0.01^klm^ | 0.39±0.01^fg^ | 0.25±0.01^ef^ |
| **Mechanical Wounding** |  |  |  |  |
| DMRE 63 | 0.22±0.01^cd^ | 0.24±0.02 ^fg^ | 0.74±0.05 ^b^ | 0.30±0.01 ^cd^ |
| CM 500 | 0.19±0.01 ^def^ | 0.16±0.01^hijk^ | 0.25±0.06 ^hijk^ | 0.26±0.02 ^def^ |
| WNZ ExoticPool | 0.25±0.01^bc^ | 0.43±0.01^c^ | 0.47±0.03 ^ef^ | 0.28±0.01^de^ |
| CM 202 | 0.17±0.01^defg^ | 0.14±0.01^ijkl^ | 0.18±0.01 ^jklm^ | 0.11±0.01^k^ |
| BML 6 | 0.14±0.01 ^efghi^ | 0.13±0.01 ^ijkl^ | 0.08±0.01^no^ | 0.20±0.01^hi^ |
| **Wounding + Regurgitation** |  |  |  |  |
| DMRE 63 | 0.14±0.01 ^efghi^ | 0.51±0.02 ^b^ | 0.18±0.02 ^jklm^ | 0.58±0.02 ^a^ |
| CM 500 | 0.13±0.01 ^fghi^ | 0.22±0.01 ^fgh^ | 0.16±0.01^klmn^ | 0.44±0.04 ^b^ |
| WNZ ExoticPool | 0.18±0.01 ^def^ | 0.32±0.01 ^d^ | 0.27±0.01 ^hij^ | 0.54±0.03^a^ |
| CM 202 | 0.1±0.06 ^ij^ | 0.19±0.01^fghi^ | 0.2±0.01^ijkl^ | 0.28±0.01^de^ |
| BML 6 | 0.16±0.01 ^efgh^ | 0.17±0.01^ghij^ | 0.21±0.01 ^ijkl^ | 0.33±0.01^c^ |
| **Exposure to methyl jasmonate** |  |  |  |  |
| DMRE 63 | 0.42±0.08 ^a^ | 0.62±0.03 ^a^ | 0.28±0.02 ^hi^ | 0.27±0.01^def^ |
| CM 500 | 0.40±0.07 ^a^ | 0.31±0.09 ^de^ | 0.1±0.01^mno^ | 0.05±0.05^l^ |
| WNZ ExoticPool | 0.19±0.02 ^de^ | 0.19±0.01 ^fghi^ | 0.62±0.02 ^cd^ | 0.23±0.01^fgh^ |
| CM 202 | 0.27±0.02 ^bc^ | 0.15±0.01^ijkl^ | 0.13±0.05 ^lmno^ | 0.12±0.01^jk^ |
| BML 6 | 0.28±0.04 ^b^ | 0.16±0.01 ^ijkl^ | 0.14±0.01 ^lmno^ | 0.09±0.01 ^kl^ |

Each value represents the mean ± SEm of 3 replications. Means within a column followed by different letters are significantly different (LSDTest p =0.05)
